# A New Antibacterial Agent-Releasing Polydimethylsiloxane Coating for Polymethyl Methacrylate Dental Restorations

**DOI:** 10.3390/jcm8111831

**Published:** 2019-11-01

**Authors:** Hang-Nga Mai, Do-Yeon Kim, Dong Choon Hyun, Ju Hayng Park, Sang Min Lee, Du-Hyeong Lee

**Affiliations:** 1 Department of Prosthodontics, School of Dentistry, Institute for Translational Research in Dentistry, Kyungpook National University, Daegu 41940, Korea; mai.hang.nga1403@gmail.com; 2 Department of Pharmacology, School of Dentistry, Kyungpook National University, Daegu 41940, Korea; dykim82@knu.ac.kr (D.-Y.K.); leeyang2324@naver.com (S.M.L.); 3 Department of Polymer Science and Engineering, Kyungpook National University, Daegu 41566, Korea; dong.hyun@knu.ac.kr (D.C.H.); pjh99279@naver.com (J.H.P.)

**Keywords:** polydimethylsiloxane, chlorhexidine, mesoporous silica nanoparticles, antibacterial coating, polymethyl methacrylate

## Abstract

Chlorhexidine (CHX) has been incorporated into the composition of polymethyl methacrylate (PMMA) dental restorations to enhance their antimicrobial performance. However, the controlled delivery of CHX remains a challenge. Although previous findings with pure silica or polymer coatings demonstrated the resistance to bacterial adhesion, they did not provide antibacterial activity beyond the coated surface. Polydimethylsiloxane (PDMS) and mesoporous silica nanoparticles (MSNs) are widely used in biomedical science as a transfer medium in drug delivery systems. Here, the MSNs are used to encapsulate CHX, and the combination is added to PDMS. A thin coating film is formed on the PMMA, using oxygen plasma and thermal treatment. The liquid chromatography analysis shows that the coating film has high encapsulation efficiency and loading capacity, with a slow and stable release rate of CHX. The cytotoxicity tests also show that the coating does not affect the proinflammatory cytokines, cellular mitotic activity, or apoptotic cell death. The ability of the coating to release CHX indicates that the coating may even be effective against bacteria that are not directly in contact with the surface. This antibacterial protective film is expected to be a novel method to inhibit bacterial activity distal to the coated surfaces of PMMA restorations.

## 1. Introduction

Polymethyl methacrylate (PMMA) is a biocompatible and nondegradable acrylic resin that has frequently been used as a restorative material in the medical and dental fields due to its ease of manipulation, acceptable aesthetics, and high cost-effectiveness [1]. However, PMMA restorations have more surface defects and higher porosity and water absorptiveness compared to metal or ceramic restorations [2,3]. In particular, when PMMA restorations are made by directly mixing a pre-polymerized powder with a liquid monomer (MMA) in the clinic, air can become trapped and develop micropores [4]. During clinical adjustment and clinical service, these micropores can be exposed on the surface due to the abrasive wear of the restorations, potentially becoming niches and incubation chambers for bacteria [5]. The presence of bacteria with pathogenic potential in the margin of the prosthesis can initiate an inflammatory response in gingival tissue [6]. Several attempts have been made to decrease the bacterial adherence and plaque accumulation on the PMMA-based restorations by adding an antibacterial agent to the composition [7,8,9,10]. Chlorhexidine (CHX) is a widely used antimicrobial agent in dentistry due to its broad spectrum of antibacterial effects [11]. Recently, in order to enhance the antibiofilm effects of CHX, mesoporous silica nanoparticles (MSNs) have been used to encapsulate the CHX [12,13]. MSNs have several advantages, including superior physicochemical properties, excellent biocompatibility, cost-effectiveness, and high loading capacity for various biomolecules, particularly due to their high porosity [14,15,16,17]. Previous reports have suggested that MSNs have the potential for clinical use as drug nanocarriers in dental biomaterials [14,18]. In a previous study, PMMA incorporated with amphotericin B-loaded MSNs was reported to provide a long-term antibacterial effect up to 14 days [18].

Since nanoparticles deliver drugs to the target site in an entrapped or immobilized form, the addition of a combination of nanoparticles and antibiotics may enhance the antibiofilm activity of PMMA restorations [19,20]. However, the direct addition of antibacterial agents to PMMA could adversely affect the mechanical properties and hinder the polymerization [8,21,22]. Alternatively, the use of a coating substance consisting of various sizes of silica nanoparticles dispersed in a methanol solvent has been shown to be an effective method for enhancing the adherence of antibacterial agents to the denture base [23]. Other recent studies have suggested the use of a 2-methacryloyloxyethyl phosphorylcholine (MPC) polymer as a coating substance, which may provide a useful modification to the PMMA surface for inhibiting plaque accumulation [1,24]. These coating substances were found to passively inhibit the adherence of early colonizers on the PMMA surface. However, their effects were limited to the physical suppression of dental plaque maturation and did not provide any active antimicrobial activity to inhibit the viability and growth of pathogenic microorganisms. 

Advanced coating substances that use an antibacterial copolymer of acrylic acid, alkyl methacrylate, and polydimethylsiloxane (PDMS) have been introduced previously [25]. PDMS is a thermally curable elastomeric polymer that has been widely used in the medical field. PDMS is compatible with diverse materials due to its fast diffusion and high bonding capacity as well as its high biocompatibility, transparency, and ease of fabrication [26,27]. Since PDMS allows the controlled release of drugs, it has been used as a transfer medium in drug delivery systems in the medical and dental fields [28]. Moreover, a PDMS polymer can be applied as a thin-film coating with a controlled thickness to protect the substrate materials and increase their hydrophobic properties [19,25,29]. 

The purpose of this article is to demonstrate the process of creating a novel CHX-releasing PDMS coating for PMMA restorations. MSNs were used to encapsulate the CHX before being delivered to PDMS, and the loading and release capacity of CHX was measured using liquid chromatography. The biological safety of the synthesized coating substance was evaluated using cytotoxicity tests.

## 2. Materials and Methods

### 2.1. Synthesis of the Nanoparticle-encapsulated Chlorhexidine (CHX@MSN)

**Figure 1:**
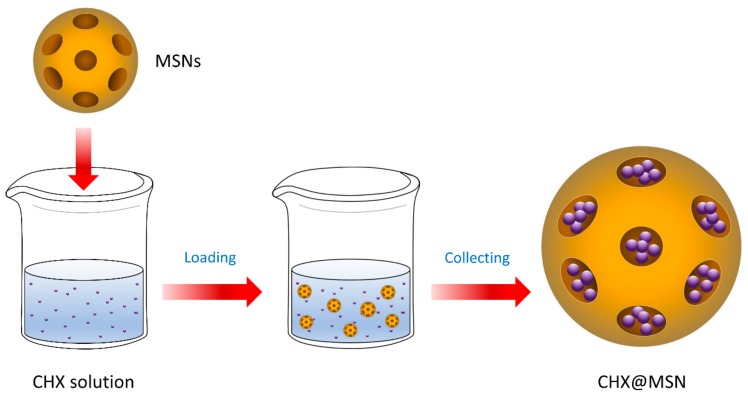
Synthesis of nanoparticle-encapsulated chlorhexidine. CHX: Chlorhexidine; MSNs: Mesoporous silica nanoparticles; CHX@MSN: MSN-encapsulated CHX.

For drug loading, 50 mg of chlorhexidine diacetate (CHX) (Sigma Aldrich, St. Louis, MO, USA) was dissolved in 5 mL of ethanol to make a CHX solution (10 mg/mL). Subsequently, 50 mg of MCM-41 mesoporous silica nanoparticles (MSN) (Sigma Aldrich, St. Louis, MO, USA) with a pore volume of 0.98 cm^3^/g and a pore size of approximately 2.5 nm was immersed in the CHX solution with physical stirring at room temperature for 24 h using a magnetic stirrer (Corning PC-420D, Thermo Fisher Scientific, Lowell, MA, USA) at a speed of 300 rpm. The MSN-encapsulated CHX (CHX@MSN) was harvested by centrifugation at 6000 rpm for 10 min using a microcentrifuge (LaboGene ScanSpeed Mini, Lynge, Denmark), and washed with ethanol and distilled water to remove the non-encapsulated CHX. After washing, the supernatant was collected for further analysis. The final CHX@MSN particles were collected after oven drying at 90 °C for 10 min using a vacuum oven (OV-11, JEIO Tech, Seoul, Korea) (Figure 1).

### 2.2. Synthesis of the CHX@MSN Coating Substance (CHX@MSN/PDMS)

**Figure 2:**
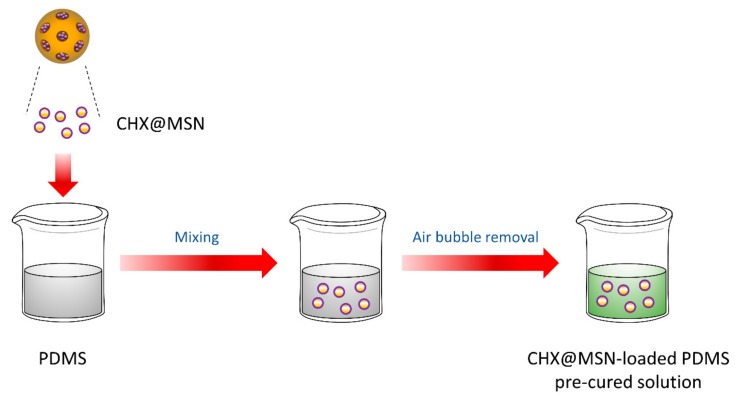
Synthesis of coating substance. PDMS: Polydimethylsiloxane.

The CHX@MSN particles were added to 3 g of polydimethylsiloxane (PDMS) (Sylgard 184, Dow Corning, Midland, MI, USA) solution in quantities of 0.0 wt %, 0.2 wt %, 0.4 wt %, and 0.6% wt % relative to the total PDMS mass. The PDMS consisted of a base and a curing agent at a weight ratio of 5:1. The mixture was stirred for 1 h using a magnetic stirrer (Corning PC-420D, Thermo Fisher Scientific, Lowell, MA, USA), ultrasonically oscillated (Wise Clean, Daehan Science, Seoul, Korea) for 30 min, and then vacuumed for 20 min to remove bubbles. The pre-cured CHX@MSN/PDMS solution was collected and divided into four groups (0 wt %; 0.2 wt %; 0.4 wt %; 0.6 wt %) according to the differences in the weight percentage of the CHX@MSN relative to the total PDMS mass. The fabrication process is illustrated in Figure 2. 

### 2.3. Coating Procedure

**Figure 3:**
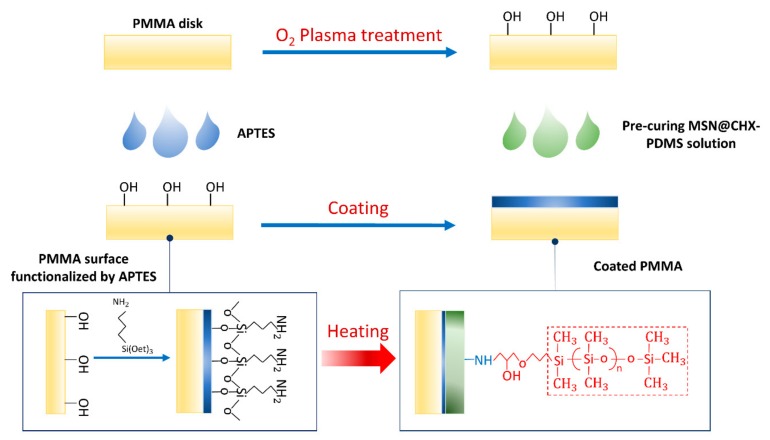
Surface functionalization and coating treatment. PMMA: Polydimethylsiloxane; APTES: 3-aminopropyltriethoxysilane.

Sixty disk-shaped resin specimens (13 mm in diameter, 1 mm in thickness) were fabricated using PMMA resin (ALIKE; GC America, Alsip, IL, USA), following the manufacturer’s instructions. The specimens were first cleaned with isopropyl alcohol (IPA) and treated by oxygen plasma (CUTE, Femto Science Co., Seoul, Korea) at a power of 70 W for 10 min. Next, the specimens were immersed in 5% (*v*/*v*) 3-aminopropyltriethoxysilane (APTES) (Sigma Aldrich, St. Louis, MO, USA) solution at 85 °C for 10 min to activate the surface of the resin specimens, forming a self-assembled layer. Subsequently, the pre-cured CHX@MSN coating solution was applied as a thin layer on the resin specimens, and the coated specimens then underwent a thermal treatment in an oven (OV-11, JEIO Tech, Seoul, Korea) at 80 °C for 2 h. The surface functionalization and coating treatment process are depicted in Figure 3.

### 2.4. Encapsulation Efficiency (EE) and Drug Loading Capacity (LC)

**Figure 4:**
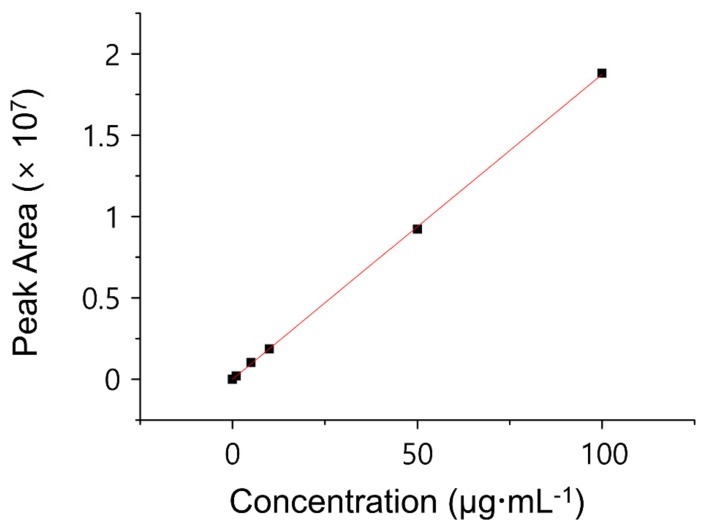
Calibration curve standard obtained from series of known concentrations of chlorhexidine.

To measure the amount of CHX in the coating substance, the percentage (%) of drug EE of the MSNs and LC of the CHX@MSN particles were determined using an indirect assay method. The amount of non-encapsulated CHX in the washed supernatant of CHX@MSN was measured using high-performance liquid chromatography (HPLC) with UV detection (Hitachi Chromaster, Hitachi, Tokyo, Japan). The chromatographic separation was performed on HPLC columns (Discovery C18, 250 mm × 4.6 mm, 5 μm, Supelco, Bellefonte, PA, USA) at 25 °C with 0.01 mol/L phosphate buffer (pH = 3.0), trimethylamine, and acetonitrile (33:66:1, *v*/*v*/*v*) as the mobile phase. The injection volume was 20 µL, and the flow rate used was 1.0 mL/min for 15 min of running time. The absorbance of UV light was detected at 239 nm wavelength (λ) using an absorbance detector. A calibration curve standard was obtained from a series of known concentrations of CHX. From the calibration graph (Figure 4), the concentration of free CHX (µg/mL) was determined using the obtained linear regression equations (*R*^2^ > 0.99). The EE and LC were calculated using Equations (1) and (2) [30]. Based on the LC and EE, Equations (3) and (4) were created to calculate the unknown percentage concentration by weight of CHX (CHX wt %) and mass of CHX ($m_{CHX}$) in the coating solution:

(1) $$\mathrm{EE}\;\left(\%\right)\;=\frac{m_{CHX_{a}}\;-\;m_{CHX_{f}}}{m_{CHX_{a}}}\times 100$$

(2) $$\mathrm{LC}\;\left(\%\right)\;=\frac{m_{CHX_{e}}}{m_{CHX@MSN}}\times 100$$

(3) $$CHX\;wt\%=\frac{m_{CHX@MSN}\;\times \;LC}{\left(m_{CHX@MSN}+m_{PDMS}\right)}$$

(4) $$m_{CHX}=\frac{CHX\;wt\%\;\times \;100^{2}}{\left(EE\;+100\right)LC}$$

where $m_{CHX_{a}}$ is the mass of the drug added, $m_{CHX_{f}}$ is the mass of the free drug after loading, $m_{CHX_{e}}$ is the mass of the encapsulated drug, $m_{CHX@MSN}$ is the mass of the nanoparticles collected after washing, and $m_{PDMS}$ is the total mass of PDMS. 

### 2.5. CHX Release Behavior

To assess the release amount of CHX, the experimental specimens were immersed in 10 mL of distilled water at pH 7.0, stored at 37 °C, and shaken at 150 rpm in a shaking incubator (Vision Scientific Co., Bucheon, Korea). Subsequently, 0.5 mL of the eluted solution (*n* = 3) was collected at the determined time points within 24 h. The cumulative release of CHX (µg/mL) from the coated PMMA disks over time was evaluated by the HPLC method using the same conditions as for EE and LC evaluation. Equations (5) and (6) were used for the calculation of the percentage of CHX release from the coating layer [31]:

(5) Concentration of drug release (µg/mL) = (Slope × absorbance) + intercept

(6) Amount of drug release (µg/mL)= Concentration × Dissolution bath volume × dilution factor.

### 2.6. Cell Culture and Drug Treatment

The NIH3T3 fibroblasts were maintained in Dulbecco’s modified Eagle’s medium (DMEM) supplemented with 10% (*v*/*v*) fetal bovine serum in a humidified atmosphere containing 5% CO_2_ at 37 °C. Eighteen hours before specimen administration or etoposide treatment, NIH3T3 cells were seeded in 12-well plates at a density of 1 × 10^5^ cells per well. For apoptosis induction, the cells were treated with 100 µM of etoposide for 24 h.

### 2.7. Quantitative Real-time RT-PCR

Total RNA was isolated from the cultured fibroblasts using an RNA Purification Kit (Thermo Fisher Scientific, Waltham, MA, USA), and the RNA was reverse-transcribed using a First Strand cDNA Synthesis Kit (Thermo Fisher Scientific, Waltham, MA, USA) according to the manufacturer’s instructions. Quantitative RT-PCR was performed on the cDNA samples using the Power SYBR Green Master Mix in the Mic qPCR Cycler (Bio Molecular Systems, Upper Coomera, Queensland, Australia). The relative mRNA levels were presented as values of 2^(Ct(β-actin) − Ct(gene of interest)). For data presentation, the mRNA levels in the control were set to 1. The sequences of the forward and reverse primers were as follows: 

β actin, 5’-GGCTGTATTCCCCTCCATCG-3’ and 5’-CCAGTTGGTAACAATGCCATGT-3’; IL-6, 5’-CCGGAGAGGAGACTTCACAG-3’ and 5’-CAGAATTGCCATTGCACAAC-3’; TNF-α, 5’-TAGCCAGGAGGGAGAACAGA-3’ and 5’-TTTTCTGGAGGGAGATGTGG-3’; IL-10, 5’-GCCTTATCGGAAATGATCCA-3’ and 5’-TCTCACCCAGGGAATTCCAAA-3’.

### 2.8. Protein Preparation and Immunoblot Analysis

Cells were disrupted directly with Laemmli buffer (60 mM Tris-HCl (pH 6.8), 2% (*w*/*v*) sodium dodecyl sulfate (SDS), 10% (*v*/*v*) glycerol, 0.02% (*w*/*v*) bromophenol blue), followed by sonication and heat denaturation at 93 °C for 5 min. For immunoblot analysis, the samples were fractionated by SDS polyacrylamide gel electrophoresis method (SDS-PAGE) and transferred to polyvinylidene fluoride (PVDF) membranes. After blocking the membranes with 5% non-fat dried milk in tris-buffered saline with Tween 20 (TBST) (10 mM Tris, pH 8.0, 150 mMNaCl, 0.5% Tween 20) for 30 min, the membranes were washed three times (10 min each) with TBST and incubated with antibodies against β-actin (1:5000, Sigma Aldrich, St. Louis, MO, USA), phospho-Histone 3 at Ser 10 (1:1000, Cell Signaling, Danvers, MA, USA), phospho-Erk1/2 at Thr202 and Tyr204 (1:1000, Thermo Fisher Scientific, Lowell, MA, USA), and phospho-AKT at Ser 473 (1:1000, Cell Signaling Technology, Danvers, MA, USA) overnight at 4 °C. The next day, the membranes were washed three times (10 min each) with TBST and incubated with horseradish peroxidase-conjugated anti-rabbit antibodies (1:5000, Bethyl Laboratories, Montgomery, TX, USA) for 1 h. The membranes were washed three times (10 min each) with TBST and the signals were detected with the D-Plus^TM^ ECL Femto System (Dongin LS, Seoul, Korea). Quantification of Western blots was performed with ImageJ 1.37c (National Institutes of Health, Bethesda, MD, USA).

### 2.9. Immunofluorescence

For immunofluorescence in cultured fibroblasts, the cells were fixed with 4% paraformaldehyde for 15 min and permeabilized with 0.2% Triton X-100 for 15 min at room temperature. After blocking the samples with 2% bovine serum albumin in phosphate-buffered saline (BSA/PBS) for 30 min, the cells were subjected to immunofluorescence staining with anti-phospho-Histone 3 at Ser 10 (1:200, Cell Signaling) and anti-cleaved caspase 3 (1:200, Cell Signaling) primary antibodies overnight at 4 °C. The next day, the cells were washed with cold PBS and incubated with Flamma 552-conjugated goat anti-rabbit IgG (BioActs, Incheon, Korea) for 30 min at room temperature. Fluorescent signals were visualized with an EVOS FL Auto Imaging System (Thermo Fisher Scientific, Waltham, MA, USA).

### 2.10. Statistical Analysis 

Statistical analysis was performed using one-way analysis of variance (ANOVA) and Fisher’s least significant difference (LSD) post hoc multiple comparison with a minimum confidence level of *p* < 0.05 for statistical significance. 

## 3. Results

### 3.1. EE and LC

The EE was 25.22%, indicating that when the ratio between CHX and MSNs was set to 1:1, and 1 mg CHX was added into the MSNs, 0.2522 mg of CHX was encapsulated in the CHX@MSN nanoparticles. The LC was determined as 63.04%, indicating that every 1 mg of CHX@MSN nanoparticles added to the coating solution contained approximately 0.63 mg CHX. Based on the LC and EE, the percentage concentrations by weight of CHX (CHX wt %) and mass of CHX ($m_{CHX}$) in the coating solution for the three groups are presented in Table 1.

### 3.2. In Vitro CHX Release Profiles

**Figure 5:**
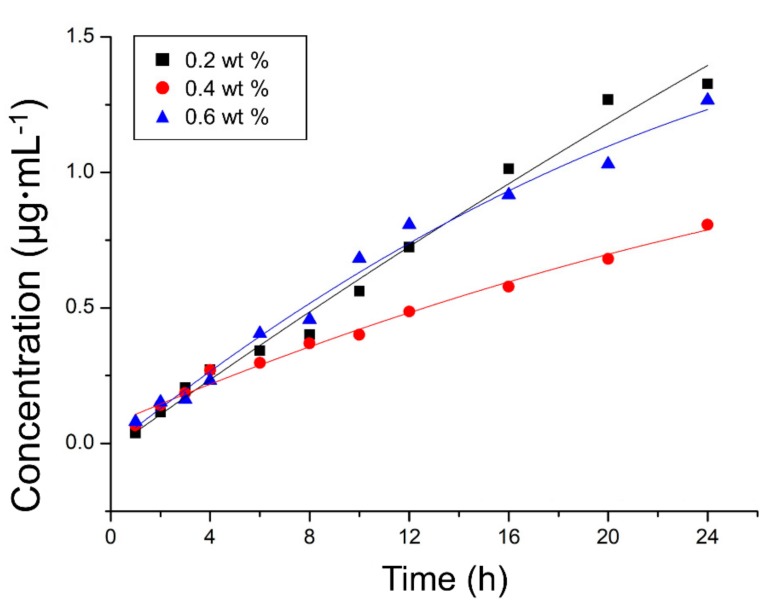
Cumulative release profiles of chlorhexidine from coated polymethyl methacrylate specimens within first 24 h.

The cumulative release profiles of CHX from the coating substance were investigated (Figure 5). The release increased rapidly during the initial period and slowed over time regardless of the specimens. Among the coated PMMA specimens containing different amounts of CHX@MSN (0.2, 0.4, or 0.6 wt %) relative to the PDMS by mass, the 0.2 wt % group exhibited the highest CHX release (up to 1.56 µg/mL), followed by the 0.6 wt % and 0.4 wt % groups (*R*^2^ = 0.98).

### 3.3. Inflammatory Cytokines

**Figure 6:**
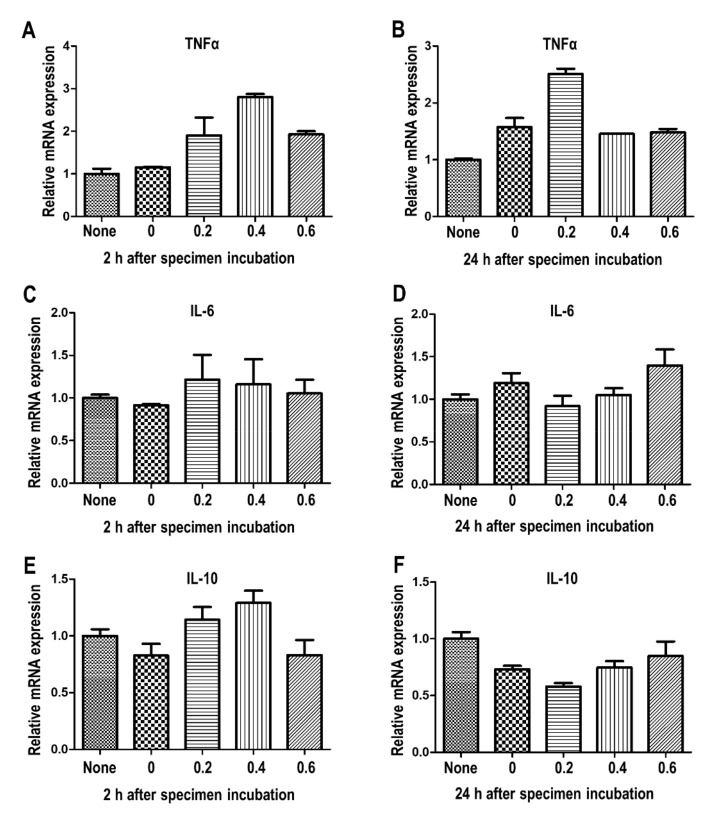
Analysis of pro- and anti-inflammatory cytokines following administration of coating material. (**A**) mRNA expression of TNF-α after 2 h, (**B**) mRNA expression of TNF-α after 24 h, (**C**) mRNA expression of IL-6 after 2 h, (**D**) mRNA expression of IL-6 after 24 h, (**E**) mRNA expression of IL-10 after 2 h, (**F**) mRNA expression of IL-10 after 24 h. The cells were subjected to quantitative RT-PCR analysis. Error bars, mean ± SEM. * Stars indicate significant differences compared to the 0 wt % group (* *p* < 0.05, ** *p* < 0.01; *** *p* < 0.001).

Cellular inflammatory response can be determined by measuring the mRNA expression of central inflammatory cytokines [32]. The mRNA expression of pro-inflammatory cytokines (interleukin (IL)-6, and tumor necrosis factor (TNF)-α) and an anti-inflammatory cytokine (IL-10) were analyzed to investigate the inflammatory reaction in the cells (Figure 6). Compared to the 0 wt %, the 0.4 wt % and 0.6 wt % of coating materials slightly upregulated the TNF-α expression within 2 h. However, the concentration of CHX release did not significantly affect the TNF-α level at 24 h, suggesting that the increased level of released CHX did not mediate the long-term TNF-α production. The data showed that the IL-6 and IL-10 expression was unaltered, regardless of the specimens, up to 24 h. 

### 3.4. Mitotic Activity

**Figure 7:**
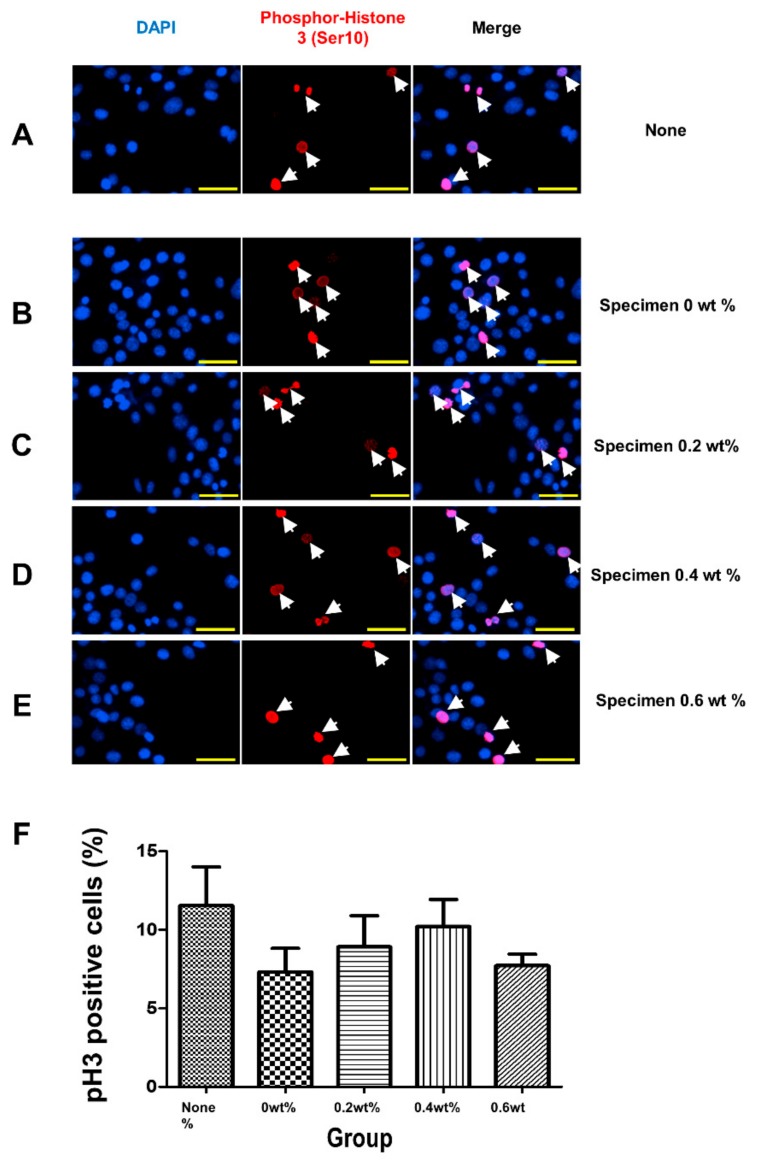
Analysis of mitotic capacity after coating material administration. (**A**–**F**) Immunofluorescence analysis of phospho-Histone 3 (Ser 10) proteins in non-treated (**A**) or coating material-challenged (**B**–**E**) cells. The concentrations of materials were indicated on the panel. Cells were incubated with specimens for 24 h. Nuclear 4′,6-diamidino-2-phenylindole staining is shown in blue. (**F**) The quantification of phospho-Histone 3 (Ser 10)-positive cell proportions was analyzed. Nuclear 4’,6-diamidino-2-phenylindole (DAPI) staining is shown in blue. The scale bar is 50 µm.

The immunofluorescence analysis was performed to detect phospho-histone 3 (ser10), a mitosis marker (Figure 7A–E). The percentages of phospho-histone 3 (ser10)-positive cells were quantified by counting random fields of view, and were found to remain unchanged regardless of the specimens (Figure 7F). 

### 3.5. Induction of Apoptotic Cell Death

**Figure 8:**
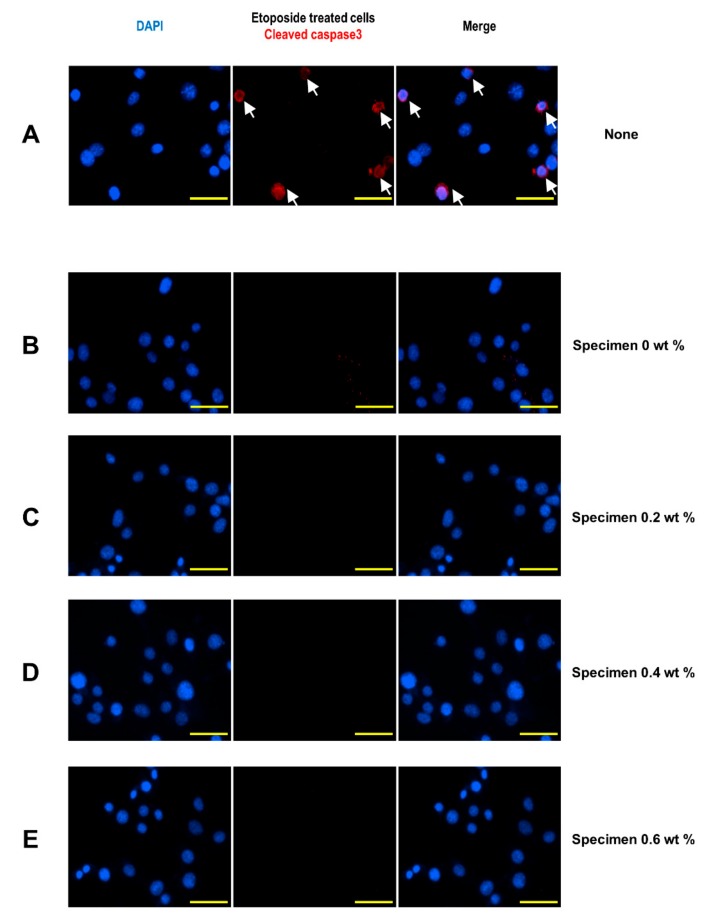
Analysis of apoptotic activity after coating material administration. (**A**) Immunofluorescence analysis of cleaved caspase 3 proteins in etoposide-treated cells. (**B**–**E**) Immunofluorescence analysis of cleaved caspase 3 proteins in specimen-treated cells. Nuclear 4’,6-diamidino-2-phenylindole staining is shown in blue. The scale bar is 50 µm.

The cleaved caspase 3 protein levels were examined with immunofluorescence analysis to evaluate the apoptotic cell death. As a positive control, etoposide, a chemotherapy drug that induces apoptosis by inhibiting DNA synthesis, was utilized, and the etoposide-treated cells clearly showed cleaved caspase 3 signals (Figure 8A). The absence of cleaved caspase 3-positive cells following the administration of specimens suggested that none of the specimens caused cytotoxic cell death (Figure 8B–E).

### 3.6. Molecular Analysis

**Figure 9:**
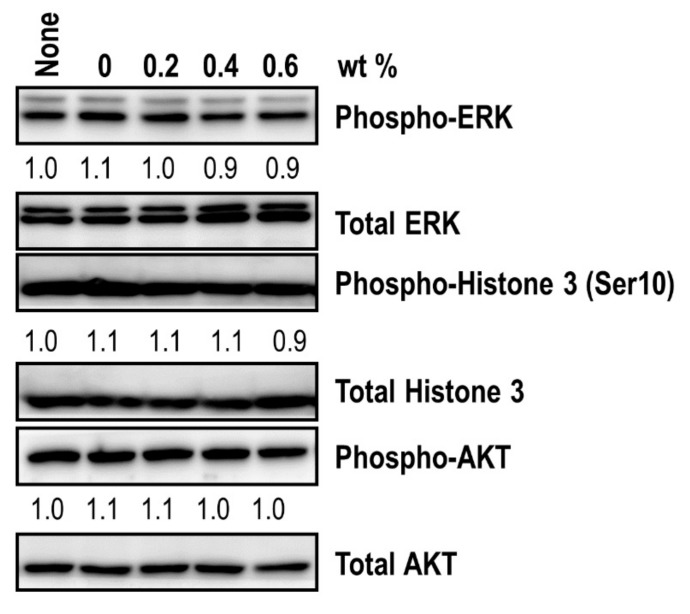
Western blot analysis of mitotic signatures following the administration of a coating material. Immunoblot analysis of phospho-ERK, phospho-histone 3 (Ser10), and phospho-AKT levels was performed after specimen introduction. The protein levels of total ERK, total histone 3, and total AKT were also measured to determine the phosphorylation level of each molecule. The relative band intensities of the phosphoproteins are shown below the bands. The sample without a specimen challenge was indicated as none. The intensity of the untreated sample was arbitrarily set to 1.

To precisely analyze the intracellular molecular events, immunoblot assays were performed (Figure 9). The specimens did not alter the phospho-ERK levels that are strongly linked to cell proliferation, suggesting that the specimens had no effect on ERK signaling activation. In addition, the levels of phospho-AKT, which promotes the survival and growth of cells, as well as the phospho-Histone 3 (ser10) levels, were unchanged, regardless of the specimens. These data confirmed that the specimens did not affect the cellular mitotic activity in general.

## 4. Discussion

The purpose of this study was to develop a new CHX-releasing coating substance using MSNs as the nanocarrier and PDMS as the host matrix. The results demonstrated that the coating layer was successfully formed on the PMMA surface and released up to 1.56 µg/mL of CHX in the first 24 h. The EE of the MSNs and LC of the CHX@MSN particles for CHX were 25.22% and 63.04%, respectively. With regard to biocompatibility, the cell viability assay clearly showed the non-cytotoxicity of the synthesized coating substance. 

In this study, CHX release was detected at a stable rate within the first 24 h. Interestingly, the CHX concentration was probably not the factor controlling the speed of CHX release during the initial period. This may be due to the use of MSNs to deliver the CHX in a PDMS host matrix. When the CHX incorporates with a hydrophobic material such as PMMA, the CHX aggregates embedded inside the resin matrix are hard to release [22,33]. Thus, the CHX release rate would decrease sharply after the first few hours, despite a large amount of CHX remaining in the matrix [21,22,33]. In contrast, PDMS has beneficial silicone materials that facilitate the adjustment of drug release patterns and improve the film formation and stability [28]. The use of MSNs as a nanocarrier for CHX may also contribute to the more sustained and controlled release of CHX over a prolonged period of time [13]. In the present study, the indirect assay demonstrated that a significant amount of CHX was capable of being loaded onto the nanoparticles by a swelling and assembly process. These results correspond well with previous studies [13,34,35]. The high EE and LC values of CHX in the present study imply that MSNs and PDMS are effective reservoirs.

In previous studies, the incorporated dosage of CHX ranged from 1 wt % to 11.5 wt % for composite resins and glass ionomer cements [21,22,33]. It has been reported that the incorporation of CHX at a high dosage results in a decrease in the mechanical properties, a decrease in the bond strength, and an increase in the setting time [21]. Moreover, despite the fact that the effects of CHX on plaque inhibition are dose-dependent, the effective dose usually ranges in the lower concentration of 0.02‒0.2% volume [36]. Solderer et al. reported that mouth rinses containing less concentrated CHX formulations showed more favorable antibiofilm formation effects because they reduced the side effects of CHX while maintaining comparable clinical effects [37]. In the present study, CHX concentrations ranging from 0.0 to 0.6 wt % were included, and the release of up to 1.56 µg/mL of CHX was higher than the minimum inhibitory concentration (MIC) of CHX for clinical isolates of *S. mutans*, which was reported to range from 0.25 µg/mL to 1 µg/mL [37,38]. The release of CHX from CHX@MSN-loaded PDMS suggests the possibility of the newly developed coating’s antibacterial activity; however, its inhibitory effects against bacterial attachment and biofilm formation under in vivo conditions remain to be determined.

The CHX@MSN and PDMS materials employed in this study have been shown to be relatively non-cytotoxic. An excessive dose of CHX could trigger cell death [39]. Since MSNs are capable of retaining CHX in the matrix, the toxicity of CHX can be lowered [12]. The results of this study showed that the concentration of CHX release did not significantly affect the pro-inflammatory cytokines TNF-α and IL-6, regardless of the specimens and release time. The TNF-α expression at 24 h was decreased compared to that at 2 h, and the IL-6 and IL-10 expression was unaltered. While IL-6 is generally known as a pro-inflammatory cytokine, it has also been reported to have an anti-inflammatory function [40]. Thus, it was thought that the synthesized layer of the specimen did not produce an inflammation response. The immunofluorescence analysis also suggested that the specimens did not affect the cellular mitotic activity in general and did not cause cytotoxic cell death. These findings indicated that CHX@MSN in PDMS not only exhibited a potential antibacterial effect but also had higher biocompatibility. 

Although this study introduced the development of a new CHX-releasing PDMS coating, as well as the promising results of CHX, there are several limitations. First, a limited period of time was used for the release profile analysis and the cell viability assay. Secondly, it was an in vitro study. The oral environment is more complex, and the composition of fluids fluctuates frequently. Accordingly, further comprehensive in vivo studies with long-term designs are needed. The physical and chemical characteristics of the new coating substance should also be investigated under relevant conditions. 

## 5. Conclusions

This study has demonstrated a novel formulation of CHX-loaded PDMS-based antibacterial coating for PMMA restorations, which are highly desirable in medical and dental applications. The results demonstrated that the coating film had a high EE and LC, with a slow and stable release rate of CHX. With regard to biocompatibility, the cell viability assay clearly showed the nontoxicity of the synthesized coating substance. Moreover, the coating substance is prepared from a simple and practical protocol that can be readily scaled up for production. This method is a promising tool likely to inspire a series of new active antibacterial functional coating materials for dental restorations.

## Figures and Tables

**Table 1:** Percentage concentration by weight of CHX (CHX wt %) and mass of CHX ($m_{CHX}$) in coating substance.

Group	$m_{CHX@MSN}\;(\mathrm{mg})$	CHX wt %	$m_{CHX}\;(\mathrm{mg})$
0 wt %	0	0.0000	0.0000
0.2 wt %	6	0.0013	0.1991
0.4 wt %	12	0.0025	0.3974
0.6 wt %	18	0.0038	0.5949

* CHX: Chlorhexidine; MSN: Mesoporous silica nanoparticle; CHX@MSN: MSN-encapsulated CHX.
